# Valor Prognóstico dos Níveis Plasmáticos de NT-proBNP em Pacientes Hospitalizados com Mais de 80 Anos de Idade em um Hospital em Pequim, China

**DOI:** 10.36660/abc.20190158

**Published:** 2021-06-08

**Authors:** Qiwei Zhu, Peng Gao, Shihui Fu, Hao Wang, Yongyi Bai, Leiming Luo, Ping Ye

**Affiliations:** 1 Chinese People’s Liberation Army General Hospital Beijing China Chinese People’s Liberation Army General Hospital , Beijing - China; 2 Department of Geriatric Cardiology Chinese People’s Liberation Army General Hospital Beijing China Department of Geriatric Cardiology , Chinese People’s Liberation Army General Hospital , Beijing - China

**Keywords:** Peptídeo Natriurético Encefálico, Prognóstico, Doença Arterial Coronariana, Hospitalização, Envelhecimento, Ecocardiografia/métodos, Hipertensão, Diabetes Mellitus, Obesidade, Idoso de 80 anos ou mais

## Abstract

**Fundamento:**

Apesar das evidências crescentes de que o peptídeo natriurético N-terminal pró-cérebro (NT-proBNP) tem um valor prognóstico importante em adultos mais velhos, há dados limitados sobre seu valor preditivo prognóstico.

**Objetivos:**

O objetivo deste estudo é avaliar o significado clínico do NT-proBNP em pacientes hospitalizados com mais de 80 anos de idade em Pequim, China.

**Métodos:**

Este estudo prospectivo e observacional foi conduzido em 724 pacientes muito idosos em uma enfermaria geriátrica (idade ≥80 anos, variação, 80-100 anos, média, 86,6±3,0 anos). A análise de regressão linear multivariada foi utilizada para rastrear os fatores independentemente associados ao NT-proBNP, e o modelo de regressão de risco proporcional de Cox foi utilizado para rastrear as associações entre os níveis de NT-proBNP e os principais *endpoints* . Os principais endpoints avaliados foram mortes por todas as causas e ECAM. Valores de p <0,05 foram considerados estatisticamente significativos.

**Resultados:**

As taxas de prevalência de doença cardíaca coronariana, hipertensão e diabetes mellitus foram 81,4%, 75,1% e 41,2%, respectivamente. O nível médio de NT-proBNP foi 770±818 pg/mL. Utilizando análises de regressão linear multivariada, foram encontradas correlações entre o NT-proBNP plasmático e índice de massa corporal, fibrilação atrial, taxa de filtração glomerular estimada, diâmetro do átrio esquerdo, fração de ejeção do ventrículo esquerdo, uso de betabloqueador, níveis de hemoglobina, albumina plasmática, triglicérides, creatinina sérica, e nitrogênio uréico no sangue. O risco de morte por todas as causas (HR, 1,63; IC 95%, 1,005-2,642; p = 0,04) e eventos cardiovasculares adversos maiores (ECAM; HR, 1,77; IC 95%, 1,289-3,531; p = 0,04) no grupo com o nível mais alto NT-proBNP foi significativamente maior do que no grupo com NT-proBNP mais baixo, de acordo com os modelos de regressão de Cox após o ajuste para vários fatores. Como esperado, os parâmetros da ecocardiografia ajustaram o valor prognóstico do NT-proBNP no modelo.

**Conclusões:**

O NT-proBNP foi identificado como um preditor independente de morte por todas as causas e ECAM em pacientes hospitalizados com mais de 80 anos de idade.

## Introdução

O peptídeo natriurético cerebral (BNP, *Brain natriuretic peptide* ) foi descrito pela primeira vez em 1988, após seu isolamento do cérebro porcino. Logo descobriu-se que o miocárdio ventricular era a principal fonte de síntese e secreção de BNP. O BNP é inicialmente sintetizado como um pré-hormônio em resposta ao estiramento do miócito, sendo então enzimaticamente clivado em BNP biologicamente ativo e o fragmento N-terminal do peptídeo natriurético tipo B (NT-ProBNP) inativo, em proporções iguais. Muitos estudos têm demonstrado que o BNP e o NT-proBNP são importantes preditores de morbidade e mortalidade cardiovascular em adultos de meia-idade e idosos. ^1 - 4^ No entanto, como há dados limitados sobre indivíduos com idade ≥80 anos, o valor preditivo do BNP e do NT-proBNP nesses idosos não está claro. ^3 , 4^

A China é o país mais populoso do mundo. Com a melhoria dos padrões de vida e das instalações médicas, a população chinesa com 80 anos ou mais aumentou gradualmente. De acordo com os resultados do censo de 2010, existem aproximadamente 20 milhões de pessoas com 80 anos ou mais na China. Como os níveis plasmáticos de NT-proBNP aumentam com a idade, mesmo na ausência de insuficiência cardíaca ou outras doenças cardiovasculares (DCV), ^5 , 6^ formulamos a hipótese de que um aumento nos níveis plasmáticos de NT-proBNP reflete o risco de morte por todas as causas e eventos cardiovasculares adversos maiores (ECAM) em indivíduos com 80 anos ou mais.

## Métodos

### População de estudo

Este estudo prospectivo e observacional examinou pacientes muito idosos (idade ≥ 80 anos) que foram hospitalizados no Departamento de Medicina Interna Geriátrica do *Chinese People’s Liberation Army (PLA) General Hospital* , Pequim, China. Os pacientes foram excluídos caso tivessem doenças sistêmicas graves, como colagenose, caquexia, infecção grave, doença hepática grave, insuficiência cardíaca aguda ou síndrome coronariana aguda, ou tivessem se submetido a cirurgia de revascularização do miocárdio ou angioplastia coronária transluminal percutânea nos 6 meses anteriores. Um total de 739 pacientes muito idosos foram incluídos entre novembro de 2007 e outubro de 2010; 326 foram hospitalizados por doença cardíaca coronária (DCC) estável, 278 foram hospitalizados por controle deficiente da pressão arterial (a pressão arterial não estava controlada dentro da faixa-alvo sem mudança do medicamento), 39 foram internados no hospital por doenças respiratórias (31 casos tinham infecções do trato respiratório superior), e 17 foram internados no hospital por doenças digestivas.

### Questionário e exame físico

As informações sobre a idade do paciente e o histórico da doença, incluindo DCC, hipertensão, fibrilação atrial (FA), diabetes mellitus (DM) e câncer, foram coletadas pelo médico na hospitalização.

O exame físico incluiu medidas de altura e peso. Após o paciente ter ficado sentado por pelo menos 5 minutos, a pressão arterial foi medida com um esfigmomanômetro de mesa calibrado, o que é consistente com as recomendações atuais. A pressão arterial do paciente foi medida três vezes consecutivamente com pelo menos 1 minuto entre as medidas, e os valores médios foram utilizados para a análise.

### Ensaio bioquímico

Todos os pacientes foram submetidos a uma avaliação laboratorial completa. Amostras de sangue foram coletadas de pacientes entre 6h e 8h após jejum noturno (≥12 horas) para medir os seguintes parâmetros: colesterol total (CT), triglicérides (TG), lipoproteína de baixa densidade-colesterol (LDL-C), lipoproteína de alta densidade-colesterol (HDL-C), creatinina sérica (CrS), nitrogênio uréico no sangue (BUN, do inglês *blood urea nitrogen* ) e NT-proBNP. As amostras de sangue foram enviadas ao Laboratório de Bioquímica do PLA General Hospital. Para cada parâmetro, os mesmos reagentes, métodos e instrumentos foram utilizados para analisar todas as amostras. As concentrações de CrS foram determinadas utilizando um ensaio enzimático (Roche Diagnostics GmbH, Basel, Suíça) e um autoanalisador Hitachi 7600 (Hitachi, Tóquio, Japão). Os níveis plasmáticos de NT-proBNP foram determinados utilizando-se um imunoensaio por eletroquimioluminescência (Roche Diagnostics GmbH, Mannheim, Alemanha) e um analisador Roche (Roche Diagnostics, Indianapolis, IN).

### Medidas da ecocardiografia

A ecocardiografia foi realizada em até 3 dias a partir da hospitalização por ultrassonografistas experientes. A fração de ejeção do ventrículo esquerdo (FEVE) foi determinada utilizando a regra de Simpson do biplano a partir de imagens cardíacas apicais de quatro e duas câmaras. ^7^ O diâmetro atrial esquerdo (DAE), o diâmetro sistólico final do ventrículo esquerdo (DSFVE), diâmetro diastólico final do ventrículo esquerdo (DDFVE), diâmetro do septo interventricular (DSI) e espessura da parede posterior (EPP) foram medidos em três batimentos consecutivos, e os resultados foram calculados como média.

### Definição das variáveis

A taxa de filtração glomerular estimada (TFGe) foi calculada utilizando a versão chinesa da equação *Modification of Diet in Renal Disease* da seguinte forma: ^8^ TFGe (mL/min/ 1,73 m ^2^ ) = 175 × CrS padrão (mg/dL) ^-1,234^ × idade (ano) ^0,179^ × 0,79 (se o paciente for do sexo feminino). A doença renal crônica (DRC) foi definida de acordo com as diretrizes da prática clínica. ^9^ O índice de massa corporal (IMC) foi definido como o peso (kg) dividido pelo quadrado da altura (m). A massa ventricular esquerda (MVE) foi calculada como {0,8 [1,04 (DDFVE + EPP + DSI) 3 - (DDFVE) ^3^ ]} + 0,6 g ^7^ . A área de superfície corporal (ASC) foi calculada como 0,0061 × altura ^0,0124^ × peso ^-0,0099^ O índice da MVE (IMVE) foi definido como a MVE dividida pela ASC. A hipertrofia ventricular esquerda (HVE) foi definida de acordo com os seguintes critérios: (i) IMVE maior que 125 g/m ^2^ (paciente masculino) e/ou (ii) IMVE> 110 g/m ^2^ (paciente feminino). ^7 , 11^ A hipertensão foi definida de acordo com os seguintes critérios: (i) pressão arterial sistólica ≥140 mm Hg, (ii) pressão arterial diastólica (PAD) ≥90 mm Hg, e/ou (iii) uso de drogas anti-hipertensivas. ^12^ A DM foi definida de acordo com os seguintes critérios: (i) níveis de glicose em jejum maiores ou iguais a 7,1 mmol/L, (ii) níveis de glicose no sangue venoso em 2 horas ≥11,1 mmol/L, e/ou (iii ) uso de hipoglicemiantes ou insulina. ^13^ Os diagnósticos de DCC, FA e câncer foram confirmados pelo histórico médico do paciente.

### Seguimento e *endpoints*

As consultas de seguimento foram realizadas de dezembro de 2015 a janeiro de 2016. Durante essas consultas no PLA *General Hospital* chinês, todos os pacientes receberam um questionário. O intervalo médio de seguimento foi de 5,3 anos [intervalo interquartil (IIQ), 2,7-6,6 anos]. Quinze pacientes foram perdidos durante o seguimento, e excluídos da análise. Dados completos de seguimento foram obtidos de 724 pacientes (taxa de seguimento, 98%).

Os principais *endpoints* avaliados foram morte por todas as causas e ECAM. A morte foi verificada a partir da certidão de óbito (um documento legal incluindo hora, local e outras informações). Os ECAM incluíram infarto do miocárdio não fatal, terapia de revascularização coronária, angina pectoris instável e hospitalização por insuficiência cardíaca ou acidente vascular cerebral. A incidência de ECAM foi o evento que não causou óbito, sendo que apenas a primeira vez foi registrada quando ocorreu mais de um evento.

### Análise estatística

O teste de Kolmogorov-Smirnov foi empregado para verificar a normalidade dos dados. As variáveis contínuas com distribuição normal foram expressas como média (± desvio padrão) e aquelas com distribuição assimétrica foram expressas como mediana e IIQ. As variáveis categóricas foram expressas em número e porcentagem. Os níveis plasmáticos de NT-proBNP sofreram transformação logarítmica natural porque não houve distribuição gaussiana. Os níveis plasmáticos de NT-proBNP basais foram categorizados como quartil 1 (≤124 pg/mL, n = 181), quartil 2 (124-271 pg/mL, n = 180), quartil 3 (271-668 pg/mL, n = 182) e quartil 4 (≥668 pg/mL, n = 181). As variáveis contínuas entre grupos foram comparadas utilizando análise de variância, enquanto a comparação entre duas amostras independentes foi realizada utilizando o teste U de Mann-Whitney. As variáveis categóricas entre os grupos foram comparadas com os testes Qui-Quadrado e Exato de Fisher.

As correlações entre as variáveis contínuas foram avaliadas através de regressão linear, e as premissas de linearidade para as variáveis independentes contínuas dos resíduos padronizados foram avaliadas através da plotagem dos resíduos contra a variável preditora, enquanto a colinearidade entre as variáveis independentes foi avaliada utilizando os fatores de inflação da variância. A análise de regressão linear multivariada (critério de entrada *P* ≤ 0,10) foi utilizada para rastrear os fatores independentemente associados ao NT-proBNP.

As relações entre os níveis de NT-proBNP e os principais *endpoints* foram avaliadas utilizando o modelo de regressão de risco proporcional de Cox. O modelo 1 foi ajustado para idade e sexo. O modelo 2 foi ajustado para as variáveis do modelo 1 mais IMC, hipertensão, FA, DCC, DM, hemoglobina, albumina plasmática, TFGe, LDL-C e HDL-C. O modelo 3 foi ajustado para variáveis do modelo 2 mais o uso de medicamentos cardiovasculares. O modelo 4 foi ajustado para as variáveis do modelo 3 mais FEVE, DAE e IMVE. Não foi utilizada correção para risco competitivo ao avaliar a relação entre NT-proBNP e ECAM. As curvas de mortalidade cumulativa e ECAM foram geradas com o método Kaplan-Meier. As curvas de características de operação do receptor (ROC) foram geradas para avaliar a precisão dos níveis de NT-proBNP na previsão de morte por todas as causas e ECAM.

Todas as análises foram realizadas com o software SPSS para Windows (versão 13.0; SPSS, Chicago, IL) e o software State (versão 11.0; Stata Corporation, College Station, TX). Valores de p <0,05 foram considerados estatisticamente significativos.

## Resultados

### Características basais dos participantes

Um total de 724 pacientes muito idosos foram incluídos na análise. A idade dos pacientes variou de 80 a 100 anos (média de 86,6 ± 3,0 anos) e a maioria dos pacientes era do sexo masculino (93,3%). Ao nível basal, o nível médio de NT-proBNP foi de 770 ± 818 pg/mL Medicamentos cardiovasculares, características demográficas, fatores de risco cardiovascular e testes laboratoriais associados em cada grupo são mostrados na Tabela 1 . Os pacientes no quartil mais alto dos níveis plasmáticos de NT-proBNP eram significativamente mais velhos, apresentavam maior prevalência de DCC e FA e tinham níveis mais altos de CrS, DAE e IMVE; esses pacientes também apresentavam um IMC mais baixo e menores níveis de TFGe, CT, TG, LDL-C, hemoglobina, albumina plasmática, FEVE e PAD.

**Tabela 1:** – Características basais dos pacientes em cada quartil de níveis plasmáticos de NT-proBNP

Características	Todos os pacientesincluídos	NT-proBNP (pg/mL)				p valor

		<124	124-271	271-668	≥668	
n	724	181	180	182	181	
Idade (anos)	86,6±3,0	85,7±2,9	86,1±2,9	87,0±2,9	87,4±3,0	<0,001
Sexo masculino (%)	680(93,9)	171(94,5)	169(93,9)	168(92,3)	172(95,0)	0,727
Sexo feminino (%)	44(6,1)	10(5,5)	11(6,1)	14(7,7)	9(5,0)	
DCC (%)	589(81,4)	135(74,6)	144(80)	150(82,4)	160(88,4)	0,008
HT (%)	544(75,1)	124(68,5)	135(75)	144(79,1)	141(77,9)	0,088
DM (%)	298(41,2)	71(39,2)	69(38,3)	76(41,8)	82(45,3)	0,537
FA (%)	130(18,0)	15(8,3)	16(8,9)	27(14,8)	72(39,8)	<0,001
IMC (kg/m ^2^ )	23,8±2,6	24,7±2,3	23,7±2,3	23,5±2,7	23,3±2,9	<0,001
NT-proBNP (pg/mL)	770±818	70±27	190±37	420±93	2399±1618	<0,001
TFGe (ml/min.1.73m ^2^ )	75,7±20,4	82,8±17,2	77,8±19,7	75,6±20,7	66,6±22,1	<0,001
CrS (ug/mL)	99,6±29,4	85,1±16,6	92,4±21,2	98,1±28,3	122,5±50,3	<0,001
BUN (mmol/L)	7,61±2,50	6,48±1,63	6,83±1,64	7,59±2,41	9,55±3,88	<0,001
CT (mmol/L)	4,14±0,67	4,31±0,67	4,24±0,65	4,00±0,69	3,99±0,61	<0,001
TG (mmol/L)	1,45±0,57	1,62±0,60	1,41±0,54	1,42±0,62	1,34±0,50	0,023
LDL-C (mmol/L)	2,37±0,57	2,54±0,59	2,46±0,57	2,23±0,57	2,26±0,52	<0,001
HDL-C (mmol/L)	1,16±0,23	1,13±0,24	1,18±0,25	1,18±0,27	1,16±0,29	0,473
Hb (g/L)	124,6±13,9	131,6±12,5	125,3±11,8	122,1±13,6	119,2±15,4	<0,001
ALB (g/L)	39±2,8	40,0±2,6	39,8±2,7	38,4±2,7	37,7±2,8	<0,001
FEVE (%)	60,0±3,8	61,4±3,1	60,8±2,9	59,7±4,2	57,9±4,4	<0,001
FEVE<40%	3	0	0	1	2	
FEVE>50%	697	180	179	173	165	
DDFVE (mm)	48,5±2,8	48,2±2,5	47,9±2,3	48,8±2,7	49,3±3,4	0,002
DSFVE (mm)	32,9±2,3	32,2±1,7	32,3±2,0	33,2±2,4	34,0±2,9	<0,001
DAE (mm)	37,2±3,1	36,3±2,7	36,0±2,8	37,4±2,6	39,3±3,9	<0,001
SIV (mm)	10,7±1,1	10,6±1,0	10,5±1,0	10,7±1,2	10,9±1,2	0,040
PPVE (mm)	10,1±0,7	10,0±0,6	10,0±0,6	10,2±0,8	10,2±0,9	0,203
IMVE (g/m ^2^ )	123,2±19,9	118,9±15,7	118,0±16,0	126,7±19,4	129,1±27,5	<0,001
Medicamento antiplaquetário (%)	493(68,1)	118(65,2)	129(71,7)	120(65,9)	126(69,6)	0,506
Estatinas (%)	311(43,0)	84(46,4)	70(38,9)	78(42,9)	79(43,6)	0,547
BCC (%)	361(49,9)	95(52,5)	93(51,7)	87(47,8)	86(47,5)	0,697
IECA (%)	92(12,7)	21(11,6)	25(13,9)	14(7,7)	32(17,7)	0,035
BRA (%)	227(31,4)	55(30,4)	62(34,4)	68(37,4)	42(23,2)	0,023
IECA/BRA (%)	307(42,4)	72(39,8)	85(47,2)	79(43,4)	71(39,2)	0,386
Betabloqueador (%)	291(40,2)	58(32,0)	56(31,1)	82(45,1)	95(52,5)	<0,001
PAS média (mmHg)	129,3±9,4	129,3±9,4	129,9±8,5	128,6±9,4	129,4±10,2	0,753
PAD média (mmHg)	67,3±5,8	68,2±5,7	68,1±5,2	66,9±5,9	66,2±6,1	0,027
PAM (mmHg)	88,0±6,0	88,6±6,2	88,7±5,2	87,5±5,9	87,3±6,4	0,152
PPmmHg)	61,9±8,6	61,1±7,9	61,9±8,7	61,7±8,6	63,2±9,2	0,348

*DCC: doença cardíaca coronária; HT: hipertensão; DM: diabetes mellitus; FA: fibrilação atrial; IMC: índice de massa corporal; NT-proBNP: peptídeo natriurético N-terminal pró-cérebro; TFGe: taxa de filtração glomerular estimada; CrS: creatinina sérica; BUN: nitrogênio uréico no sangue; AU: ácido úrico; CT: colesterol total; TG: triglicérides; LDL-C: lipoproteína de baixa densidade-colesterol; HDL-C: lipoproteína de alta densidade-colesterol; Hb: hemoglobina; ALB: albumina plasmática; FEVE: fração de ejeção do ventrículo esquerdo; DDFVE: diâmetro diastólico final do ventrículo esquerdo; DSFVE: diâmetro sistólico final do ventrículo esquerdo; DAE: diâmetro do átrio esquerdo; SIV: septo interventricular; PPVE: parede posterior do ventrículo esquerdo; IMVE: índice de massa ventricular esquerda; BCC: bloqueador dos canais de cálcio; IECA: inibidor da enzima conversora de angiotensina; BRA: bloqueadores dos receptores da angiotensina; PAS: pressão arterial sistólica; PAD: pressão arterial diastólica; PAM: pressão arterial média; PP: pressão de pulso.*

### Associação de níveis plasmáticos de NT-proBNP com variáveis clínicas

Ao nível basal, a idade avançada, DCC, FA, CrS, BUN, DAE e IMVE foram positivamente associados aos níveis plasmáticos de NT-proBNP, enquanto a TFGe, CT, LDL-C, TG, hemoglobina, albumina plasmática, FEVE, IMC, PAD, e a pressão arterial média foram inversamente associados aos níveis plasmáticos de NT-proBNP, como demonstrado pelos resultados das análises univariadas. Utilizando a análise de regressão linear multivariada, a idade avançada (p = 0,019), FA, CrS, BUN, DAE e o uso de betabloqueador foram positivamente associados com os níveis plasmáticos de NT-proBNP, enquanto a TFGe, TG, hemoglobina, albumina plasmática, FEVE, e o IMC foram inversamente associados aos níveis plasmáticos de NT-proBNP ( Tabela 2 ).

**Tabela 2:** – Associação dos níveis plasmáticos de NT-proBNP com variáveis clínicas

Variáveis	Univariada		Multivariada		

	Valor de r	Valor de p	Valor β padrão	Valor de p	95% IC
Sexo	0,003	0,926			
Idade	0,178	<0,001	0,082	0,019	0,002 to 0,023
DCC	0,136	<0,001	-0,004	0,913	-0,104 to 0,093
HT	0,072	0,053	0,011	0,753	-0,075 to 0,104
FA	0,310	<0,001	0,218	0,000	0,213 to 0,414
DM	0,047	0,202			
TFGe	-0,240	<0,001	-0,131	0,003	-0,005 to 0,000
Crs	0,285	<0,001	0,192	0,001	0,001 to 0,003
BUN	0,325	<0,001	0,112	0,010	0,004 to 0,028
CT	-0,162	<0,001	0,058	0,485	-0,067 to 0,142
LDL-C	-0,173	<0,001	-0,050	0,535	-0,161 to 0,084
HDL-C	0,026	0,495			
TG	-0,111	0,004	-0,088	0,018	-0,107 to -0,010
Hb	-0,293	<0,001	-0,121	0,002	-0,006 to -0,001
ALB	-0,287	<0,001	-0,137	0,000	-0,033 to -0,009
FEVE	-0,261	<0,001	-0,179	0,000	-0,029 to -0,013
DAE	0,292	<0,001	0,179	0,000	0,015 to 0,036
IMVE	0,163	<0,001	0,006	0,865	-0,001 to 0,001
IMC	-0,170	<0,001	-0,111	0,005	-0,032 to -0,006
Droga antiplaquetária	0,026	0,478			
Estatinas	0,007	0,848			
BCC	-0,056	0,136			
BRA	-0,047	0,204			
IECA	0,074	0,046	0,057	0,088	-0,014 to 0,205
IECA/BRA	0,005	0,883			
Betabloqueador	0,172	<0,001	0,124	0,000	0,066 to 0,219
PAS	-0,007	0,860			
PAD	-0,110	0,003	0,000	0,992	-0,006 to 0,005
PAM	-0,075	0,042			
PP	0,065	0,080	0,065	0,064	0,000 to 0,007

*DCC: doença cardíaca coronária; HT: hipertensão; DM: diabetes mellitus; FA: fibrilação atrial; IMC: índice de massa corporal; NT-proBNP: peptídeo natriurético N-terminal pró-cérebro; TFGe: taxa de filtração glomerular estimada; Crs: creatinina sérica; BUN: nitrogênio uréico no sangue; CT: colesteróis totais; TG: triglicérides; LDL-C: lipoproteína de baixa densidade-colesterol; HDL-C: lipoproteína de alta densidade-colesterol; Hb: hemoglobina; ALB: albumina plasmática; FEVE: fração de ejeção do ventrículo esquerdo; DAE: diâmetro do átrio esquerdo; IMVE: índice de massa ventricular esquerda; BCC: bloqueador dos canais de cálcio; IECA: inibidor da enzima conversora de angiotensina; BRA: bloqueadores dos receptores da angiotensina; PAS: pressão arterial sistólica; PAD: pressão arterial diastólica; PAM: pressão arterial média; PP: pressão de pulso.*

### Associação de níveis plasmáticos de NT-proBNP com mortalidade por todas as causas e ECAM

**Figura 1:**
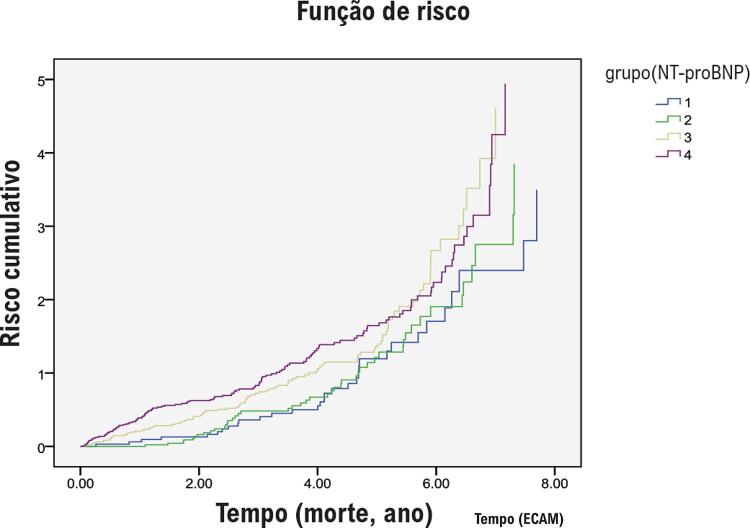
– Curvas de Kaplan-Meier demonstrando a incidência cumulativa de morte por todas as causas em pacientes muito idosos com diferentes níveis de NT-proBNP (quartil 1: <124 pg/mL, quartil 2: 124-271 pg/mL, quartil 3: 271-668 pg/mL e quartil 4: ≥668 pg/mL). O risco de mortalidade por todas as causas foi significativamente maior no quartil 4 (77,3%) do que no quartil 1 (28,7%) (HR = 1,63; IC 95%, 1,005-2,642; p = 0,04). Log-rank, p = 0,008. NT-proBNP: peptídeo natriurético N-terminal pró-cérebro; HR: Hazard Ratio; IC: intervalo de confiança.

Durante um seguimento médio de 5,3 anos (IIQ 2,7–6,6 anos), 353 pacientes (48,8%) morreram; 45 (12,7%) morreram de causas cardíacas e 150 (42,5%) morreram de infecção. A taxa de mortalidade por todas as causas aumentou significativamente de 28,7% no quartil mais baixo dos níveis plasmáticos de NT-proBNP (<124 pg/mL) para 77,3% no quartil mais alto dos níveis plasmáticos de NT-proBNP (≥668 pg/mL), de acordo com os resultados utilizando um modelo não ajustado. A análise de sobrevida de Kaplan-Meier foi realizada para estudar a relação entre os subgrupos e a probabilidade de sobrevida; os pacientes com níveis mais elevados de NT-proBNP apresentaram uma probabilidade de sobrevida significativamente menor ( *P* = 0,008; Figura 1 ). O risco de morte por todas as causas [ *Hazard Ratio* (HR), 1,63; Intervalo de confiança de 95% (IC), 1,005–2,642; *P* = 0,04)] para pacientes no quartil mais alto dos níveis plasmáticos de NT-proBNP foi significativamente maior do que para pacientes no quartil mais baixo dos níveis plasmáticos de NT-proBNP, de acordo com os resultados utilizando o modelo de regressão de risco proporcional de Cox após o ajuste para idade, sexo, IMC, presença de uma comorbidade (HT, DCC ou FA), TFGe, pressão de pulso, uso de um medicamento cardiovascular (IECA e betabloqueador) e níveis de BUN, TG, hemoglobina e albumina plasmática (Modelo 3; Tabela 3 ).

**Tabela 3:** – Associação dos níveis plasmáticos de NT-proBNP com morte, ECAM, SCA e acidente vascular cerebral

HR (IC95%)				

	Grupo 1	Grupo 2	Grupo 3	Grupo 4

	(n=181)	(n=180)	(n=182)	(n=181)
Mortalidade por todas as causas	52(28,7%)	60(33,3%)	101(55,5%)	140(77,3%)
HR não ajustado	1(controle)	1,058(0,729-1,535)	1,301(0,928-1,822)	1,432(1,039-1,974)
Modelo 1	1(controle)	1,073(0,686-1,680)	1,52(1,020-2,265)	1,668(1,137-2,449)
Modelo 2	1(controle)	1,057(0,629-1,777)	1,414(0,864-2,312)	1,583(0,984-2,545)
Modelo 3	1(controle)	0,993(0,583-1,687)	1,391(0,847-2,285)	1,629(1,005-2,642)
Modelo 4	1(controle)	0,934(0,549-1,590)	1,354(0,819-2,236)	1,473(0,884-2,454)
ECAM	30(16,6%)	41(22,8%)	49(26,9%)	82(45,3%)
HR não ajustado	1(controle)	0,512(0,309-0,849)	0,516(0,322-0,827)	0,568(0,370-0,874)
Modelo 1	1(controle)	1,020(0,569-1,828)	1,025(0,591-1,778)	1,979(1,193-3,285)
Modelo 2	1(controle)	1,021(0,492-2,118)	0,975(0,508-1,873)	1,748(0,893-3,425)
Modelo 3	1(controle)	0,956(0,446-2,053)	1,071(0,545-2,102)	1,769(1,289-3,531)
Modelo 4	1(controle)	0,799(0,362-1,762)	0,797(0,392-1,621)	1,313(0,621-2,780)
SCA	16(53,3%)	25(61%)	34(69,4%)	62(75,6%)
HR não ajustado	1(controle)	1,55(0,86-2,78)	1,74(0,97-3,10)	2,02(1,33-3,59)
Modelo 1	1(controle)	1,53(0,85-2,76)	1,67(0,93-2,99)	2,01(1,25-3,58
Modelo 2	1(controle)	2,04(0,99-4,17)	1,48(0,72-3,04)	2,12(1,18-4,45)
Modelo 3	1(controle)	1,94(0,94-4,01)	1,39(0,66-2,92)	1,89(1,14-4,08)
Modelo 4	1(controle)	1,67(0,81-3,47)	1,12(0,51-2,44)	1,54(0,87-3,58)
AVC	12(40%)	11(26,8%)	7(14,3%)	6(7,3%)
HR não ajustado	1(controle)	0,66(0,31-1,37)	0,27(0,11-0,71)	0,39(0,15-1,01)
Modelo 1	1(controle)	0,737(0,35-1,56)	0,25(0,10-0,64)	0,41(0,16-1,08)
Modelo 2	1(controle)	1,36(0,48-3,77)	0,28(0,09-0,84)	0,59(0,17-2,03)
Modelo 3	1(controle)	1,27(0,44-3,68)	0,32(0,12-1,01)	0,73(0,19-2,80)
Modelo 4	1(controle)	1,28(0,40-4,10)	0,34(0,09-1,26)	0,93(0,21-4,23)

*O modelo 1 foi ajustado para idade e sexo. O modelo 2 foi ajustado para as variáveis do modelo 1 mais hipertensão, DM, FA, DCC, IMC, hemoglobina, albumina plasmática, TFGe, LDL-C e HDL-C. O modelo 3 foi ajustado para as variáveis do modelo 2 mais medicamentos cardiovasculares. O modelo 4 foi ajustado para as variáveis do modelo 3 mais FEVE, DAE e IMVE. NT-proBNP: peptídeo natriurético N-terminal pró-cérebro; SCA: síndrome coronariana aguda; HR: Hazard Ratio; IC: intervalo de confiança; DCC: doença cardíaca coronária; HT: hipertensão; DM: diabetes mellitus; FA: fibrilação atrial; IMC: índice de massa corporal; LDL-C: lipoproteína de baixa densidade-colesterol; HDL-C: lipoproteína de alta densidade-colesterol; TFGe: taxa de filtração glomerular estimada; FEVE: fração de ejeção do ventrículo esquerdo; DAE: diâmetro do átrio esquerdo; IMVE: índice de massa ventricular esquerda; ECAM: eventos cardiovasculares adversos maiores.*

**Figura 2:**
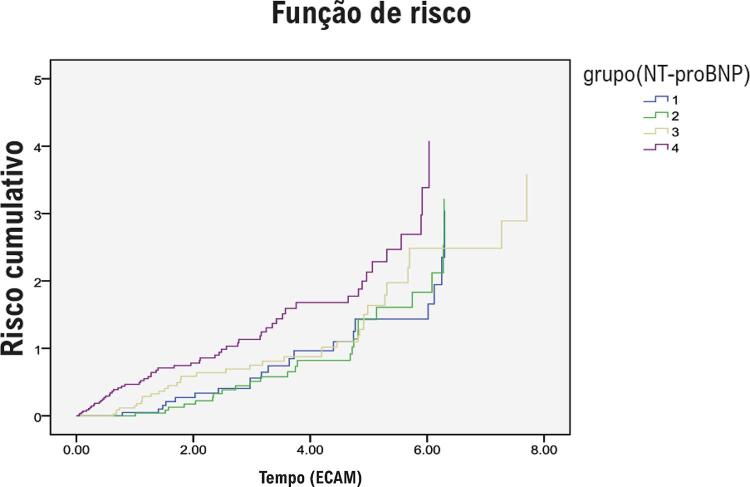
– Curvas de Kaplan-Meier demonstrando a incidência cumulativa de ECAM em pacientes muito idosos com diferentes níveis de NT-proBNP (quartil 1: <124 pg/mL, quartil 2: 124-271 pg/mL, quartil 3: 271-668 pg/mL e quartil 4: ≥668 pg/mL). O risco de ECAM foi significativamente maior no quartil 4 (45,3%) do que no quartil 1 (16,6%) (HR = 1,77; IC de 95%, 1,289-3,531; p = 0,04). Log-rank, p = 0,002. NT-proBNP: peptídeo natriurético N-terminal pró-cérebro; HR: Hazard Ratio; IC: intervalo de confiança.

Havia 202 pacientes com ECAM durante o seguimento. A incidência de ECAM aumentou significativamente de 16,6% no quartil mais baixo dos níveis plasmáticos de NT-proBNP para 45,3% no quartil mais alto dos níveis plasmáticos de NT-proBNP. Uma análise de sobrevida de Kaplan-Meier revelou diferenças significativas entre os grupos (teste *log-rank* , p = 0,002; Figura 2 ). O risco de ECAM (HR, 1,77; IC95%, 1,29-3,53; p = 0,04) para pacientes no quartil mais alto dos níveis plasmáticos de NT-proBNP foi significativamente maior do que para pacientes no quartil mais baixo dos níveis plasmáticos de NT-proBNP, após o ajuste para múltiplos fatores de risco cardiovascular. Uma análise adicional de subgrupo encontrou que a maior incidência de ECAM foi a síndrome coronariana aguda (SCA) não fatal (67,8%). O risco de SCA (HR, 1,89; IC95%, 1,14-4,08; p = 0,04) para pacientes no quartil mais alto dos níveis plasmáticos de NT-proBNP foi significativamente maior do que para pacientes no quartil mais baixo dos níveis plasmáticos de NT-proBNP após ajuste para múltiplos fatores de risco cardiovascular (Modelo 3). Entretanto, os níveis plasmáticos de NT-proBNP não foram associados ao risco de morte (HR, 1,47; IC95%, 0,88-2,45; p = 0,14), ECAM (HR, 1,31; IC95%, 0,62-2,78; p = 0,48) ou SCA (HR, 1,54; IC95%, 0,87-3,58; p = 0,20), de acordo com os resultados obtidos utilizando o modelo de regressão de risco proporcional de Cox após ajuste adicional para FEVE, DAE e IMVE (Modelo 4; Tabela 3 ).

### Curvas ROC dos níveis plasmáticos de NT-proBNP para prever morte por todas as causas e ECAM

**Figura 3:**
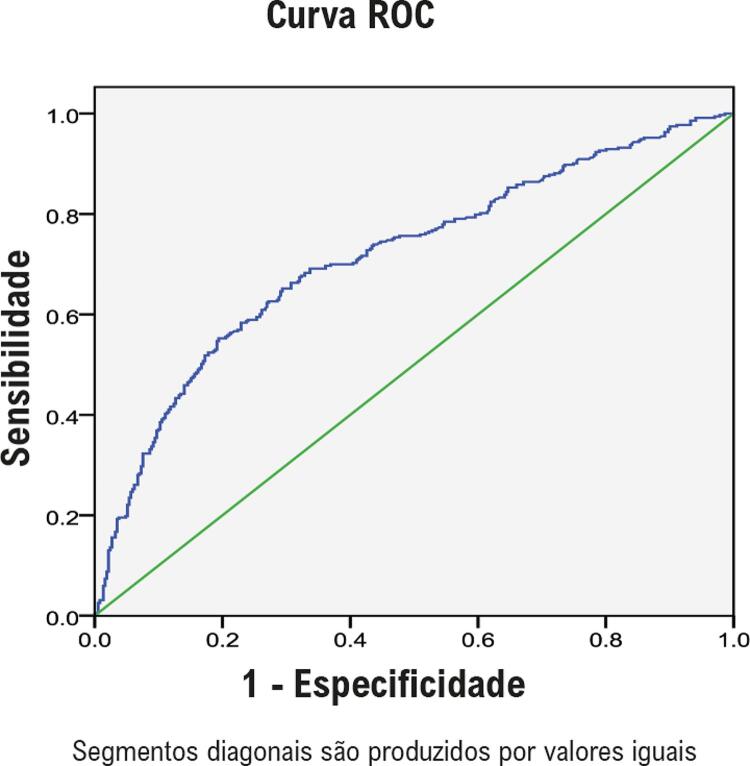
Curva ROC de NT-proBNP para prever a morte por todas as causas. A AUC foi de 0,71 (IC de 95%, 0,677-0,752), p <0,001. ROC: receiver operating characteristic; NT-proBNP: peptídeo natriurético N-terminal pró-cérebro; AUC: área sob a curva; IC: intervalo de confiança.

**Figura 4:**
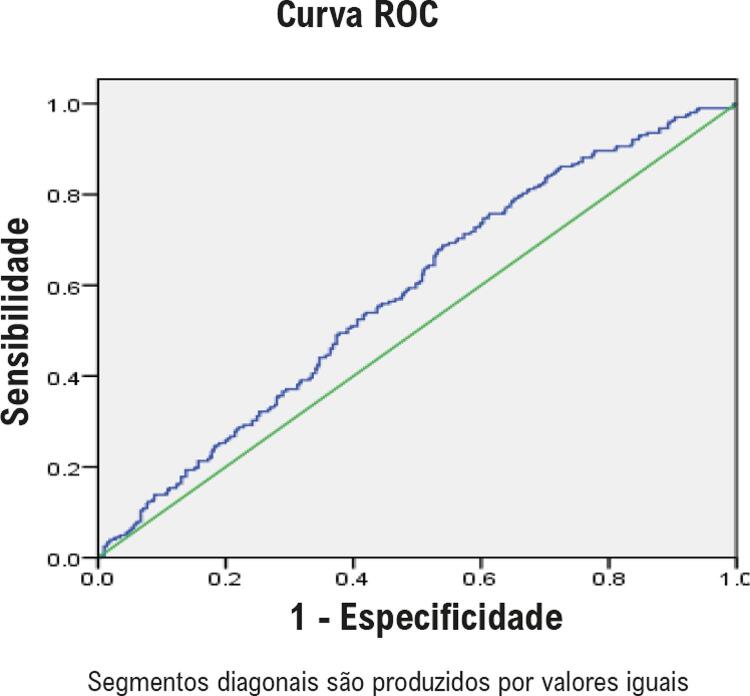
– Curva ROC de NT-proBNP para prever os ECAM. A AUC foi de 0,58 (IC de 95%, 0,537-0,626), p = 0,001. ROC: receiver operating characteristic; NT-proBNP: peptídeo natriurético N-terminal pró-cérebro; AUC: área sob a curva; IC: intervalo de confiança.

Os dados apresentados nas curvas ROC mostram que o NT-proBNP é um preditor razoavelmente preciso de morte por todas as causas e ECAM. A área sob a curva ROC foi de 0,71 (IC95%, 0,677–0,752; p<0,001) para morte por todas as causas ( Figura 3 ). O valor de corte para os níveis plasmáticos de NT-proBNP para prever morte por todas as causas foi de 406 pg/mL e mostrou um índice de Youden máximo de 0,36, com uma sensibilidade de 65% e uma especificidade de 81%. A área sob a curva ROC foi de 0,58 (IC95%, 0,537–0,626; p=0,001) para ECAM ( Figura 4 ). O valor de corte para os níveis plasmáticos de NT-proBNP para prever ECAM foi de 406 pg/mL e mostrou um índice de Youden máximo de 0,23, com uma sensibilidade de 69% e uma especificidade de 54%.

## Discussão

O principal achado deste estudo é que o NT-proBNP é um preditor independente de morte por todas as causas e ECAM nessa população muito idosa, embora os parâmetros da ecocardiografia tenham enfraquecido seu valor preditivo. Além disso, o risco de morte (77,3%) e ECAM em 5 anos (45,3%) estava particularmente aumentado em indivíduos com níveis plasmáticos de NT-proBNP ≥668 pg/mL neste estudo, sugerindo que é possível ter uma avaliação de risco independente com NT-proBNP em pacientes idosos.

Muitos estudos confirmaram que o NT-proBNP é um biomarcador preditivo importante em diferentes populações, ^1^ não apenas em pacientes com insuficiência cardíaca e outras DCV, ^14 , 15^ mas também na população geral. ^2 , 16^ No entanto, em pessoas muito idosas, há dados limitados sobre seu valor preditivo prognóstico. No presente estudo, o NT-proBNP foi um preditor independente de morte por todas as causas e ECAM em pacientes muito idosos (≥80 anos), o que é consistente com os resultados de estudos anteriores. ^4^ Vaes et al. ^4^ relataram pela primeira vez que o NT-proBNP era um fator prognóstico independente em idosos ( > 85 anos). Entretanto, para aquela população específica, o histórico de DCV foi baseado em diferentes padrões de diagnóstico; nem todos os participantes realizaram exame de ecocardiografia. ^4^ Esses fatores podem afetar o valor prognóstico do NT-proBNP.

No presente estudo, os históricos de hipertensão, DCC e FA foram baseados em padrões de diagnóstico aceitos; todos os participantes foram submetidos a um exame de ecocardiografia. O NT-proBNP foi um preditor independente de morte por todas as causas, ECAM e SCA após ajuste para idade, sexo e fatores de risco cardiovascular tradicionais. Uma vez que o valor prognóstico do NT-proBNP não estava mais presente significativamente após o ajuste para os parâmetros ecocardiográficos (FEVE, DAE e IMVE), nossa hipótese é que a medida do NT-proBNP e os achados da ecocardiografia podem se complementar. A medida do NT-proBNP é uma forma rápida e barata de possivelmente evitar a necessidade de um ecocardiograma no caso de valores baixos e, por outro lado, é uma melhor indicação para um ecocardiograma nos casos de nível de NT-proBNP mais alto.

Atualmente, poucos estudos têm discutido o valor prognóstico do NT-proBNP em idosos e não há pesquisas sobre o valor de corte ideal para os níveis plasmáticos de NT-proBNP para predizer morte ou ECAM nessa população.

Em estudos anteriores, os valores de corte ideais diferiram para diferentes populações, ^17 , 18^ os quais foram muito altos em pacientes com insuficiência cardíaca aguda descompensada ^19 , 20^ e <90 pg/mL na população geral. ^16 , 18^ Fu et al., ^17^ relataram que o valor de corte ideal para NT-proBNP para prever a morte em pacientes chineses mais velhos com doença arterial coronariana é de 369,5 pg/mL em pacientes sem DRC e 2.584,1 pg/mL em pacientes com DRC. Neste estudo, os resultados das curvas ROC indicam que o NT-proBNP é um preditor razoavelmente preciso para morte por todas as causas e ECAM. As áreas sob as curvas ROC foram 0,71 (IC95%, 0,677-0,752) para morte por todas as causas e 0,58 (IC95% CI, 0,537-0,626) para ECAM. O valor de corte para os níveis plasmáticos de NT-proBNP (406 pg/mL) apresentou uma sensibilidade de 65% e uma especificidade de 81% para prever a morte por todas as causas, e uma sensibilidade de 69% e uma especificidade de 54% para prever ECAM. Mas este valor não é adequado como o valor de corte ideal para prever a morte por todas as causas e ECAM devido à baixa especificidade e sensibilidade. Simultaneamente, também foi observado que os indivíduos no quartil mais alto (nível de NT-proBNP ≥ 668pg/mL) apresentou 77,3% de risco de morte e 45,3% de risco de ECAM durante o período de seguimento, significativamente maior do que nos outros três grupos; isso identifica uma população de alto risco, e é clinicamente relevante.

Acreditamos que é possível ter uma avaliação de risco independente ao avaliar os níveis de NT-proBNP nesses pacientes idosos. Foi muito semelhante ao aumento do risco de morbidade e mortalidade cardiovascular observado por van Peet et al., ^6^ encontrado nos tercis mais elevados dos níveis de NT-proBNP para homens (nível de corte 649pg/mL), bem como nos tercis superiores dos níveis de NT-proBNP para mulheres (nível de corte 519pg/mL). Eles declararam que altos níveis de NT-proBNP podem ajudar os médicos a identificar pacientes que provavelmente irão se beneficiar mais com o seguimento proativo, e nossos resultados foram consistentes com os deles.

Este estudo tem várias limitações. Primeiro, apenas 45 (12,7%) pacientes morreram de causas cardíacas neste estudo; a maioria morreu de falência múltipla de órgãos. Portanto, o valor preditivo do NT-proBNP para morte cardíaca não foi analisado neste estudo. Em segundo lugar, embora os resultados tenham sido ajustados para múltiplas covariáveis que podem estar associadas aos níveis plasmáticos de NT-proBNP, é possível que fatores de confusão residuais, como tumores, implantação de marca-passo e isquemia miocárdica silenciosa possam impactar os achados. Terceiro, devido ao longo período de seguimento, os medicamentos cardiovasculares primários utilizados podem ter mudado com o tempo e, portanto, podem não ter refletido nos resultados deste estudo. Quarto, este estudo foi realizado em apenas um centro na China, a população consistia quase exclusivamente de homens e todos os pacientes estavam hospitalizados e eram muito idosos, portanto, os resultados não podem ser aplicados a uma população mais ampla. Quinto, a fragilidade e outros parâmetros físicos não foram avaliados neste estudo, o que pode impactar os resultados. Em sexto lugar, a análise das incidências de ECAM não considerou um modelo de risco concorrente com morte não cardíaca como risco concorrente, o que pode ter subestimado o valor prognóstico do NT-proBNP para prever ECAM.

## Conclusão

O NT-proBNP foi identificado como um preditor independente de morte por todas as causas e ECAM em pacientes hospitalizados com mais de 80 anos de idade.

## References

[1] 1. Santaguida PL, Don-Wauchope AC, Oremus M, McKelvie R, Ali U, Hill SA, et al. BNP and NT-proBNP as prognostic markers in persons with acute decompensated heart failure: a systematic review. Heart Fail Rev. 2014; 19(4): 453–70. 10.1007/s10741-014-9442-y 25062653

[2] 2. Wang TJ, Larson MG, Levy D, Benjamin EJ, Leip EP, Omland T, et al. Plasma natriuretic peptide levels and the risk of cardiovascular events and death. N Engl J Med. 2004;350(7):655–63. 10.1056/NEJMoa031994 14960742

[3] 3. Poortvliet R, de Craen A, Gussekloo J, de Ruijter W. Increase in N-terminal pro-brain natriuretic peptide levels, renal function and cardiac disease in the oldest old. Age Ageing. 2015; 44(5):841-7. 10.1093/ageing/afv091 26209786

[4] 4. Vaes B, de Ruijter W, Degryse J, Westendorp RG, Gussekloo J. Clinical relevance of a raised plasma N-terminal pro-brain natriuretic peptide level in a population-based cohort of nonagenarians. J Am Geriatr Soc. 2009; 57(5):823-9. 10.1111/j.1532-5415.2009.02218.x 19470010

[5] 5. Van Vliet P, Sabayan B, Wijsman LW, Poortvliet RK, Mooijaart SP, de Ruijter W, et al. NT-proBNP, blood pressure, and cognitive decline in the oldest old: The Leiden 85-plus Study. Neurology. 2014; 83(13):1192-9. 10.1212/WNL.0000000000000820 PMC4176025 25142900

[6] 6. Van Peet PG, de Craen AJ, Gussekloo J, de Ruijter W. Plasma NT-proBNP as predictor of change in functional status, cardiovascular morbidity and mortality in the oldest old: the Leiden 85-plus study. Age (Dordr) .2014; 36(3):9660. 10.1007/s11357-014-9660-1 PMC4082596 24807464

[7] 7. Lang RM, Bierig M, Devereux RB, Flachskampf FA, Foster E, Pellikka PA, et al. Recommendations for chamber quantification: a report from the American Society of Echocardiography’s Guidelines and Standards Committee and the Chamber Quantification Writing Group, developed in conjunction with the European Association of Echocardiography, a branch of the European Society of Cardiology. J Am Soc Echocardiogr. 2005; 18(12):1440–63. 10.1016/j.echo.2005.10.005 16376782

[8] 8. Ma YC, Zuo L, Chen JH, Luo Q, Yu XQ, Li Y, et al. Modified glomerular filtration rate estimating equation for Chinese patients with chronic kidney disease. J Am Soc Nephrol. 2006; 17(10): 2937–44. 10.1681/ASN.2006040368 16988059

[9] 9. [kidney Disease: Improving Global Outcomes(KDIGO) CKD Work Group]. KIDGO 2012 clinical practice guideline for the evaluation and management of chronic kidney disease. Kidney Int Supplements. 2013; 3(1):1-150.

[10] 10. Hu YM, Wu XL, Hu ZH, Ren AH, Wei XQ, Wang XC, et al. Research on the formula of human body surface area in China. Journal of Physiology. 1999; 51(1):45-8. 11972174

[11] 11. Mancia G, Laurent S, Agabiti-Rosei E, Ambrosioni E, Burnier M, Caulfield MJ, et al. Reappraisal of European guidelines on hypertension management: a European Society of Hypertension Task Force document. J Hypertens. 2009; 27(11):2121-58. 10.1097/HJH.0b013e328333146d 19838131

[12] 12. Chobanian AV, Bakris GL, Black HR, Cushman WC, Green LA, Izzo JL Jr, et al. Seventh report of the Joint National Committee on prevention, detection, evaluation, and treatment of high blood pressure. Hypertension. 2003; 42(6): 1206–52. 10.1161/01.HYP.0000107251.49515.c2 14656957

[13] 13. Hu J, Wallace DC, Jones E, Liu H. Cardiometabolic health of Chinese older adults with diabetes living in Beijing, China. Public Health Nurs. 2009; 26(6): 500–11. 10.1111/j.1525-1446.2009.00810.x 19903270

[14] 14. Maisel AS, Krishnaswamy P, Nowak RM, McCord J, Hollander JE, Duc P, et al. Rapid measurement of B-type natriuretic peptide in the emergency diagnosis of heart failure. N Engl J Med. 2002; 347(3):161–7. 10.1056/NEJMoa020233 12124404

[15] 15. Kragelund C, Gronning B, Kober L, Hildebrandt P, Steffensen R. N-terminal pro-B-type natriuretic peptide and long-term mortality in stable coronary heart disease. N Engl J Med. 2005; 352(7):666–75. 10.1056/NEJMoa042330 15716560

[16] 16. Zhu Q, Xiao W, Bai Y, Ye P, Luo L, Gao P, et al. The prognostic value of the plasma N-terminal pro-brain natriuretic peptide level on all-cause death and major cardiovascular events in a community-based population. Clin Interv Aging. 2016; 11:245—53. 10.2147/CIA.S98151 PMC4777226 27013868

[17] 17. Fu S, Luo L, Ye P, Yi S, Liu Y, Zhu B, et al. The ability of NT-proBNP to detect chronic heart failure and predict all-cause mortality is higher in older Chinese coronary artery disease patients with chronic kidney disease. Clin Interv Aging. 2013; 8:409-17. 10.2147/CIA.S42700 PMC3665499 23723693

[18] 18. Linssen GC, Bakker SJ, Voors AA, Gansevoort RT, Hillege HL, de Jong PE, et al. N-terminal pro-B-type natriuretic peptide is an independent predictor of cardiovascular morbidity and mortality in the general population. European Heart Journal. 2010; 31(1):120-7. 10.1093/eurheartj/ehp420 19854731

[19] 19. Wei BQ, Yang YJ, Zhang J, Dou KF, Zhang YH, Huang XH, et al. Predictive value of admission amino-terminal pro-B-type natriuretic peptide on in-hospital mortality in patients with decompensated heart failure. Zhonghua Xin Xue Guan Bing Za Zhi. 2009; 37(6):481-5. 19927625

[20] 20. Martín Sánchez FJ, Covarrubias M, Terán C, Llorens P, Herrero P, Jacob J, et al. Prognostic role of NT-proBNP in emergency department in the older with acute heart failure. Rev Esp Geriatr Gerontol. 2013; 48(4):155-60. 10.1016/j.regg.2012.11.010 23528263

