# Supplementary material for: Valor Prognóstico dos Níveis Plasmáticos de NT-proBNP em Pacientes Hospitalizados com Mais de 80 Anos de Idade em um Hospital em Pequim, China
Source: Arq Bras Cardiol. 2021 Jun 8;116(6):1027–36. [Article in Portuguese] doi: 10.36660/abc.20190158 (PMC8288545; doi:10.36660/abc.20190158)
Supplement: Translation

# Translation: Prognostic Value of Plasma NT-proBNP levels in Hospitalized Patients Older than 80 Years of Age in a Hospital in Beijing, China

**Authors:** Qiwei Zhu, Peng Gao, Shihui Fu, Hao Wang, Yongyi Bai, Leiming Luo, Ping Ye

**Affiliations:** 1 China Chinese People’s Liberation Army General Hospital, Beijing - China; 2 China Department of Geriatric Cardiology, Chinese People’s Liberation Army General Hospital, Beijing - China

**Keywords:** Natriuretic Peptide Brain, Prognosis, Coronary Artery Disease, Hospitalization, Aging; Echocardiography/methods, Hypertension, Diabetes Mellitus, Aged 80 and over

## Abstract

**Background:**

Despite growing evidence that N-terminal pro-brain natriuretic peptide (NT-proBNP) has an important prognostic value in older adults, there is limited data on its prognostic predictive value.

**Objectives:**

The aim of this study is to evaluate the clinical significance of NT-proBNP in hospitalized patients older than 80 years of age in Beijing, China.

**Methods:**

This prospective, observational study was conducted in 724 very elderly patients in a geriatric ward (age ≥80 years, range, 80100 years, mean, 86.6 3.0 years). Multivariate linear regression analysis was used to screen for factors independently associated with NT-proBNP, and the Cox proportional hazard regression model was used to screen for relationships between NT-proBNP levels and major endpoints. The major endpoints assessed were all-cause death and MACEs. P values < 0.05 were considered statistically significant.

**Results:**

The prevalence rates of coronary heart disease, hypertension, and diabetes mellitus were 81.4%, 75.1%, and 41.2%, respectively. The mean NT-proBNP level was 770 ± 818 pg/mL. Using multivariate linear regression analyses, correlations were found between plasma NT-proBNP and body mass index, atrial fibrillation, estimated glomerular filtration rate, left atrial diameter, left ventricular ejection fraction, use of betablocker, levels of hemoglobin, plasma albumin, triglycerides, serum creatinine, and blood urea nitrogen. The risk of all-cause death (HR, 1.63; 95% CI, 1.0052.642; P = 0.04) and major adverse cardiovascular events (MACE; HR, 1.77; 95% CI, 1.2893.531; P = 0.04) in the group with the highest NT-proBNP level was significantly higher than that in the group with the lowest level, according to Cox regression models after adjusting for multiple factors. As expected, echocardiography parameters adjusted the prognostic value of NT-proBNP in the model.

**Conclusions:**

NT-proBNP was identified as an independent predictor of all-cause death and MACE in hospitalized patients older than 80 years of age.

## Introduction

Brain natriuretic peptide (BNP) was first described in 1988 after its isolation from porcine brain. The ventricular myocardium was soon found to be the major source of BNP synthesis and secretion. BNP is initially synthesized as a prehormone in response to myocyte stretch and then it is enzymatically cleaved into biologically active BNP and biologically inactive N-terminal pro-BNP (NT-proBNP), in equal proportions. Many studies have shown that BNP and NT-proBNP are important predictors of cardiovascular morbidity and mortality in middle-aged and older adults.^1 - 4^ However, as there are limited data on individuals aged ≥80 years, the predictive value of BNP and NT-proBNP in such elderly individuals is unclear.^3 , 4^

China is the most populous country in the world. With improvements in living standards and medical facilities, the Chinese population aged 80 years and older has gradually increased. According to the results of the 2010 census, there are approximately 20 million people aged 80 years and older in China. As plasma NT-proBNP levels increase with age, even in the absence of heart failure or other cardiovascular diseases (CVD),^5 , 6^ we hypothesized that an increase in plasma NT-proBNP levels reflects the risk of all-cause death and major adverse cardiovascular events (MACE) in those aged 80 years and older.

## Methods

### Study population

This prospective, observational study examined very elderly patients (age ≥ 80 years) who were hospitalized in the Department of Geriatric Internal Medicine at the Chinese People’s Liberation Army (PLA) General Hospital, Beijing, China. Patients were excluded if they had severe systemic diseases, such as collagenosis, cachexia, severe infection, severe liver disease, acute heart failure, or acute coronary syndrome, or had undergone coronary artery bypass grafting or percutaneous transluminal coronary angioplasty in the previous 6 months. A total of 739 very elderly patients were enrolled between November 2007 and October 2010; 326 were hospitalized for stable coronary heart disease (CHD), 278 were hospitalized for poor blood pressure control (blood pressure was not controlled within the target range with unchanged drug treatment), 39 were admitted to the hospital for respiratory diseases (31 cases were upper respiratory tract infections), and 17 were admitted to the hospital for digestive diseases.

### Questionnaire and physical examination

Information about patient age and disease history, including CHD, hypertension, atrial fibrillation (AF), diabetes mellitus (DM), and cancer, was collected by the physician upon admission to the hospital.

The physical examination included measurements of height and weight. After the patient had been seated for at least 5 minutes, blood pressure was measured using a calibrated desktop sphygmomanometer, which is consistent with current recommendations. The patient’s blood pressure was measured three times consecutively with at least 1 minute between measurements, and the mean values were used for the analysis.

### Biochemical assay

All patients underwent a complete laboratory evaluation. Blood samples were collected from patients between 6 am and 8 am after overnight fasting (≥12 hours) to measure the following parameters: total cholesterol (TC), triglycerides (TG), low-density lipoprotein cholesterol (LDL-C), high-density lipoprotein cholesterol (HDL-C), serum creatinine (sCr), blood urea nitrogen (BUN), and NT-proBNP. Blood samples were sent to the Biochemical Laboratory of the General Hospital of the PLA. For each parameter, the same reagents, methods, and instruments were used to analyze all samples. Concentrations of sCr were determined using an enzymatic assay (Roche Diagnostics GmbH, Basel, Switzerland) and a Hitachi 7600 autoanalyzer (Hitachi, Tokyo, Japan). Plasma NT-proBNP levels were determined using an electrochemiluminescence immunoassay (Roche Diagnostics GmbH, Mannheim, Germany) and a Roche analyzer (Roche Diagnostics, Indianapolis, IN).

### Echocardiography measurements

Echocardiography was performed within 3 days of admission by experienced ultrasonographists. Left ventricular ejection fraction (LVEF) was determined using the biplane Simpson’s rule from apical four- and two-chamber images of the heart.^7^ Left atrial diameter (LAD), left ventricular end-systolic diameter, left ventricular end-diastolic diameter (LVEDd), interventricular septal diameter (IVSd), and posterior wall thickness (PWT) were measured on three consecutive beats, and the results were averaged.

### Variable definition

Estimated glomerular filtration rate (eGFR) was calculated using the Chinese version of the Modification of Diet in Renal Disease equation as follows:^8^ eGFR (mL/min/1.73 m^2^) = 175 × standard sCr (mg/dL)^1^× age (year)^0^× 0.79 (if female). Chronic kidney disease (CKD) was defined according to clinical practice guidelines.^9^ Body mass index (BMI) was defined as weight (kg) divided by the square of the height (m). Left ventricular mass (LVM) was calculated as {0.8 [1.04 (LVEDd + PWT + IVSd)^3^– (LVEDd)^3^]} + 0.6 g^7^. Body surface area (BSA) was calculated as 0.0061 × height^0^× weight^-0^.^10^ LVM index (LVMI) was defined as LVM divided by BSA. Left ventricular hypertrophy (LVH) was defined according to the following criteria: (i) LVMI greater than 125 g/m^2^(male) and/or (ii) LVMI > 110 g/m^2^(female).^7 , 11^ Hypertension was defined according to the following criteria: (i) systolic blood pressure greater than or equal to 140 mm Hg, (ii) diastolic blood pressure (DBP) greater than or equal to 90 mm Hg, and/or (iii) the use of antihypertensive drugs.^12^ DM was defined according to the following criteria: (i) fasting glucose levels greater than or equal to 7.1 mmol/L, (ii) 2-h venous blood glucose levels greater than or equal to 11.1 mmol/L, and/or (iii) the use of hypoglycemic drugs or insulin.^13^ Diagnoses of CHD, AF, and cancer were confirmed by the patient’s medical history.

### Follow-up and Endpoints

The follow-up visits were conducted from December 2015 to January 2016. During these visits to the Chinese PLA General Hospital, all patients received a questionnaire. The median follow-up interval was 5.3 years [Interquartile range (IQR), 2.7-6.6 years]. During the follow-up, 15 patients were lost and excluded from the analysis. Complete follow up data were obtained from 724 patients (follow-up rate, 98%).

The major endpoints assessed were all-cause death and MACEs. Death was ascertained from the death record (a legal document including time, site, and other information). The MACE included non-fatal myocardial infarction, coronary revascularization therapy, unstable angina pectoris, and hospitalization for heart failure or stroke. The incidence of MACE was the event that did not cause death, and only the first time was recorded when more than one occurred.

### Statistical analyses

The Kolmogorov-Smirnov test employed to verify to the normality of the data. Continuous variables with a normal distribution were expressed as the mean (± standard deviation), and those with a skewed distribution were expressed as the median and IQR. Categorical variables were expressed as the number and percentage. Plasma NT-proBNP levels underwent natural logarithmic transformation because there was no Gaussian distribution. Plasma NT-proBNP levels at baseline were categorized as quartile 1 (≤124 pg/mL, n = 181), quartile 2 (124–271 pg/mL, n = 180), quartile 3 (271–668 pg/mL, n = 182), and quartile 4 (≥668 pg/mL, n = 181). Continuous variables between groups were compared using analysis of variance, whereas comparison between two independent samples was performed using Mann-Whitney U test. The categorical variables between groups were compared using the Chi-Square and Fisher’s exact tests.

The correlations between continuous variables were assessed using linear regression and the assumptions of linearity for the continuous independent variables of the standardized residuals were assessed by plotting the residuals against a predictor variable, whereas collinearity between the independent variables was evaluated using the variance inflation factors. The multivariate linear regression analysis (entry criteria p ≤ 0.10) was used to screen the factors independently associated with NT-proBNP.

The relationships between NT-proBNP levels and major endpoints were evaluated using Cox proportional hazard regression model. Model 1 was adjusted for age and gender. Model 2 was adjusted for the variables in model 1 plus BMI, hypertension, AF, CHD, DM, hemoglobin, plasma albumin, eGFR, LDL-C, and HDL-C. Model 3 was adjusted for the variables in model 2 plus the use of cardiovascular drugs. Model 4 was adjusted for the variables in model 3 plus LVEF, LAD, and LVMI. A correction for competing risk was not used when evaluating the relationship between NT-proBNP and MACE. Cumulative mortality and MACE curves were generated using the Kaplan-Meier method. Receiver operating characteristic (ROC) curves were generated to evaluate the accuracy of NT-proBNP levels in the prediction of all-cause death and MACE.

All analyses were conducted using SPSS software for Windows (version 13.0; SPSS, Chicago, IL) and State software (version 11.0; Stata Corporation, College Station, TX). *P* values < 0.05 were considered statistically significant.

## Results

### Baseline characteristics of participants

A total of 724 very elderly patients were included in the analysis. Patients’ ages ranged from 80 to 100 years (mean, 86.6 ± 3.0 years) and the majority of patients were males (93.3%). At baseline, the mean NT-proBNP level was 770 ± 818 pg/mL. Cardiovascular drugs, demographic characteristics, cardiovascular risk factors, and related laboratory tests in each group are shown in Table 1 . Patients in the highest quartile of plasma NT-proBNP levels were significantly older, had a higher prevalence of CHD and AF, and had higher levels of sCr, LAD, and LVMI; these patients also had a lower BMI and lower levels of eGFR, TC, TG, LDL-C, hemoglobin, plasma albumin, LVEF, and DBP.

**Table 1:** – Baseline characteristics of patients in each quartile of plasma NT-proBNP levels

Characteristics	All patients included	NT-proBNP (pg/mL)				p value

		<124	124-271	271-668	≥668	
n	724	181	180	182	181	
Age(years)	86.6±3.0	85.7±2.9	86.1±2.9	87.0±2.9	87.4±3.0	<0.001
Male(%)	680(93.9)	171(94.5)	169(93.9)	168(92.3)	172(95.0)	0.727
Female(%)	44(6.1)	10(5.5)	11(6.1)	14(7.7)	9(5.0)	
CHD(%)	589(81.4)	135(74.6)	144(80)	150(82.4)	160(88.4)	0.008
HT(%)	544(75.1)	124(68.5)	135(75)	144(79.1)	141(77.9)	0.088
DM(%)	298(41.2)	71(39.2)	69(38.3)	76(41.8)	82(45.3)	0.537
AF(%)	130(18.0)	15(8.3)	16(8.9)	27(14.8)	72(39.8)	<0.001
BMI(kg/m^2^)	23.8±2.6	24.7±2.3	23.7±2.3	23.5±2.7	23.3±2.9	<0.001
NT-proBNP(pg/mL)	770±818	70±27	190±37	420±93	2399±1618	<0.001
eGFR(ml/min.1.73m^2^)	75.7±20.4	82.8±17.2	77.8±19.7	75.6±20.7	66.6±22.1	<0.001
sCr(ug/mL)	99.6±29.4	85.1±16.6	92.4±21.2	98.1±28.3	122.5±50.3	<0.001
BUN(mmol/L)	7.61±2.50	6.48±1.63	6.83±1.64	7.59±2.41	9.55±3.88	<0.001
TC(mmol/L)	4.14±0.67	4.31±0.67	4.24±0.65	4.00±0.69	3.99±0.61	<0.001
TG(mmol/L)	1.45±0.57	1.62±0.60	1.41±0.54	1.42±0.62	1.34±0.50	0.023
LDL-C(mmol/L)	2.37±0.57	2.54±0.59	2.46±0.57	2.23±0.57	2.26±0.52	<0.001
HDL-C(mmol/L)	1.16±0.23	1.13±0.24	1.18±0.25	1.18±0.27	1.16±0.29	0.473
Hb(g/L)	124.6±13.9	131.6±12.5	125.3±11.8	122.1±13.6	119.2±15.4	<0.001
ALB(g/L)	39±2.8	40.0±2.6	39.8±2.7	38.4±2.7	37.7±2.8	<0.001
LVEF(%)	60.0±3.8	61.4±3.1	60.8±2.9	59.7±4.2	57.9±4.4	<0.001
LVEF<40%	3	0	0	1	2	
LVEF>50%	697	180	179	173	165	
LVEDD(mm)	48.5±2.8	48.2±2.5	47.9±2.3	48.8±2.7	49.3±3.4	0.002
LVESD(mm)	32.9±2.3	32.2±1.7	32.3±2.0	33.2±2.4	34.0±2.9	<0.001
LAD(mm)	37.2±3.1	36.3±2.7	36.0±2.8	37.4±2.6	39.3±3.9	<0.001
IVS(mm)	10.7±1.1	10.6±1.0	10.5±1.0	10.7±1.2	10.9±1.2	0.040
PLVW(mm)	10.1±0.7	10.0±0.6	10.0±0.6	10.2±0.8	10.2±0.9	0.203
LVMI(g/m^2^)	123.2±19.9	118.9±15.7	118.0±16.0	126.7±19.4	129.1±27.5	<0.001
Anti-platelet drugs(%)	493(68.1)	118(65.2)	129(71.7)	120(65.9)	126(69.6)	0.506
Statins(%)	311(43.0)	84(46.4)	70(38.9)	78(42.9)	79(43.6)	0.547
CCB(%)	361(49.9)	95(52.5)	93(51.7)	87(47.8)	86(47.5)	0.697
ACEI(%)	92(12.7)	21(11.6)	25(13.9)	14(7.7)	32(17.7)	0.035
ARB(%)	227(31.4)	55(30.4)	62(34.4)	68(37.4)	42(23.2)	0.023
ACEI/ARB(%)	307(42.4)	72(39.8)	85(47.2)	79(43.4)	71(39.2)	0.386
Betablocker(%)	291(40.2)	58(32.0)	56(31.1)	82(45.1)	95(52.5)	<0.001
Mean SBP(mmHg)	129.3±9.4	129.3±9.4	129.9±8.5	128.6±9.4	129.4±10.2	0.753
Mean DBP(mmHg)	67.3±5.8	68.2±5.7	68.1±5.2	66.9±5.9	66.2±6.1	0.027
MBP(mmHg)	88.0±6.0	88.6±6.2	88.7±5.2	87.5±5.9	87.3±6.4	0.152
PP(mmHg)	61.9±8.6	61.1±7.9	61.9±8.7	61.7±8.6	63.2±9.2	0.348

*CHD: coronary heart disease; HT: hypertension; DM: diabetes mellitus; AF: atrial fibrillation; BMI: body mass index; NT-proBNP: N-terminal pro-brain natriuretic peptide; eGFR: estimated glomerular filtration rate; sCr: serum creatinine; BUN: blood urea nitrogen; UA: uric acid; TC: total cholesterols; TG: triglycerides; LDL-C: low density lipoprotein-cholesterol; HDL-C: high density lipoprotein-cholesterol; Hb: hemoglobin; ALB: plasma albumin; LVEF: left ventricular ejection fraction; LVEDD: left ventricular end-diastolic diameter; LVESD: left ventricular end-systolic diameter; LAD: left atrial diameter; IVS: interventricular septum; PLVM: posterior left ventricular wall; LVMI: left ventricular mass index; CCB: calcium channel blocker; ACEI: angiotensin converting enzyme inhibitor; ARB: angiotensin receptor antagonists; SBP: systolic blood pressure; DBP: diastolic blood pressure; MBP: mean blood pressure; PP: pulse pressure.*

### Association of plasma NT-proBNP levels with clinical variables

At baseline, older age, CHD, AF, sCr, BUN, LAD, and LVMI were positively associated with plasma NT-proBNP levels, whereas eGFR, TC, LDL-C, TG, hemoglobin, plasma albumin, LVEF, BMI, DBP, and mean blood pressure levels were inversely associated with plasma NT-proBNP levels, as shown by the results of the univariate analyses. Using the multivariate linear regression analysis, older age (p = 0.019), AF, sCr, BUN, LAD, and using a betablocker were positively associated with plasma NT-proBNP levels, whereas eGFR, TG, hemoglobin, plasma albumin, LVEF, and BMI were inversely associated with plasma NT-proBNP levels ( Table 2 ).

**Table 2:** – Association of plasma NT-proBNP levels with clinical variables

Variables	Univariate		Multivariate		

	r value	p value	Standard β value	p value	95% CI
sex	0.003	0.926			
Age	0.178	<0.001	0.082	0.019	0.002 to 0.023
CHD	0.136	<0.001	-0.004	0.913	-0.104 to 0.093
HT	0.072	0.053	0.011	0.753	-0.075 to 0.104
AF	0.310	<0.001	0.218	0.000	0.213 to 0.414
DM	0.047	0.202			
eGFR	-0.240	<0.001	-0.131	0.003	-0.005 to 0.000
sCr	0.285	<0.001	0.192	0.001	0.001 to 0.003
BUN	0.325	<0.001	0.112	0.010	0.004 to 0.028
TC	-0.162	<0.001	0.058	0.485	-0.067 to 0.142
LDL-C	-0.173	<0.001	-0.050	0.535	-0.161 to 0.084
HDL-C	0.026	0.495			
TG	-0.111	0.004	-0.088	0.018	-0.107 to -0.010
Hb	-0.293	<0.001	-0.121	0.002	-0.006 to -0.001
ALB	-0.287	<0.001	-0.137	0.000	-0.033 to -0.009
LVEF	-0.261	<0.001	-0.179	0.000	-0.029 to -0.013
LAD	0.292	<0.001	0.179	0.000	0.015 to 0.036
LVMI	0.163	<0.001	0.006	0.865	-0.001 to 0.001
BMI	-0.170	<0.001	-0.111	0.005	-0.032 to -0.006
Antiplatelet drug	0.026	0.478			
statins	0.007	0.848			
CCB	-0.056	0.136			
ARB	-0.047	0.204			
ACEI	0.074	0.046	0.057	0.088	-0.014 to 0.205
ACEI/ARB	0.005	0.883			
Beta-blocker	0.172	<0.001	0.124	0.000	0.066 to 0.219
SBP	-0.007	0.860			
DBP	-0.110	0.003	0.000	0.992	-0.006 to 0.005
MBP	-0.075	0.042			
PP	0.065	0.080	0.065	0.064	0.000 to 0.007

*CHD: coronary heart disease; HT: hypertension; DM: diabetes mellitus; AF: atrial fibrillation; BMI: body mass index; NT-proBNP: N-terminal pro-brain natriuretic peptide; eGFR: estimated glomerular filtration rate; sCr: serum creatinine; BUN: blood urea nitrogen; TC: total cholesterols; TG: triglyceride; LDL-C: low density lipoprotein cholesterol; HDL-C: high density lipoprotein cholesterol; Hb: hemoglobin; ALB: plasma albumin; LVEF: left ventricular ejection fraction; LAD: left atrial diameter; LVMI: left ventricular mass index; CCB: calcium channel blocker; ACEI: angiotensin converting enzyme inhibitor; ARB: angiotensin receptor antagonists; SBP: systolic blood pressure; DBP: diastolic blood pressure; MBP: mean blood pressure; PP: pulse pressure.*

### Association of plasma NT-proBNP levels with all-cause mortality and MACE

During a median follow-up of 5.3 years (IQR 2.7–6.6 years), 353 patients (48.8%) died; 45 (12.7%) died from cardiac causes and 150 (42.5%) died from an infection. The all-cause mortality rate significantly increased from 28.7% in the lowest quartile of plasma NT-proBNP levels (<124 pg/mL) to 77.3% in the highest quartile of plasma NT-proBNP levels (≥668 pg/mL), according to results using an unadjusted model. A Kaplan-Meier survival analysis was performed to study the relationship between the subgroups and survival probability; patients with higher NT-proBNP levels had a significant lower survival probability (p = 0.008; Figure 1 ). All-cause death risk [hazard ratio (HR), 1.63; 95% confidence interval (CI), 1.005–2.642; p = 0.04)] for patients in the highest quartile of plasma NT-proBNP levels was significantly higher than that for patients in the lowest quartile of plasma NT-proBNP levels, according to results using the Cox proportional hazard regression model after adjusting for age, gender, BMI, presence of a comorbidity (HT, CHD, or AF), eGFR, pulse pressure, use of a cardiovascular drug (ACEI and betablocker), and levels of BUN, TG, hemoglobin, and plasma albumin (Model 3; Table 3 ).

**Figure 1:** – Kaplan-Meier curves demonstrating the cumulative incidence of all-cause death in the very elderly with different NT-proBNP levels (quartile 1: <124 pg/mL, quartile 2: 124-271 pg/mL, quartile 3: 271-668 pg/mL, and quartile 4: ≥668 pg/mL). The risk of all-cause mortality was significantly higher in quartile 4 (77.3%) than that in quartile 1 (28.7%) (HR=1.63; 95%CI, 1.005-2.642; p=0.04). Log-rank, p=0.008. Abbreviations: NT-proBNP: N-terminal pro-brain natriuretic peptide; HR: hazard ratio; CI: confidence interval.

**Table 3:** – Association of plasma NT-proBNP levels with death, MACE, ACS and stroke

HR (95% CI)				

	Group 1	Group2	Group 3	Group 4
	
	(n=181)	(n=180)	(n=182)	(n=181)
All-cause mortality	52(28.7%)	60(33.3%)	101(55.5%)	140(77.3%)
Unadjusted HR	1(control)	1.058(0.729-1.535)	1.301(0.928-1.822)	1.432(1.039-1.974)
Model 1	1(control)	1.073(0.686-1.680)	1.52(1.020-2.265)	1.668(1.137-2.449)
Model 2	1(control)	1.057(0.629-1.777)	1.414(0.864-2.312)	1.583(0.984-2.545)
Model 3	1(control)	0.993(0.583-1.687)	1.391(0.847-2.285)	1.629(1.005-2.642)
Model 4	1(control)	0.934(0.549-1.590)	1.354(0.819-2.236)	1.473(0.884-2.454)
MACE	30(16.6%)	41(22.8%)	49(26.9%)	82(45.3%)
Unadjusted HR	1(control)	0.512(0.309-0.849)	0.516(0.322-0.827)	0.568(0.370-0.874)
Model 1	1(control)	1.020(0.569-1.828)	1.025(0.591-1.778)	1.979(1.193-3.285)
Model 2	1(control)	1.021(0.492-2.118)	0.975(0.508-1.873)	1.748(0.893-3.425)
Model 3	1(control)	0.956(0.446-2.053)	1.071(0.545-2.102)	1.769(1.289-3.531)
Model 4	1(control)	0.799(0.362-1.762)	0.797(0.392-1.621)	1.313(0.621-2.780)
ACS	16(53.3%)	25(61%)	34(69.4%)	62(75.6%)
Unadjusted HR	1(control)	1.55(0.86-2.78)	1.74(0.97-3.10)	2.02(1.33-3.59)
Model 1	1(control)	1.53(0.85-2.76)	1.67(0.93-2.99)	2.01(1.25-3.58
Model 2	1(control)	2.04(0.99-4.17)	1.48(0.72-3.04)	2.12(1.18-4.45)
Model 3	1(control)	1.94(0.94-4.01)	1.39(0.66-2.92)	1.89(1.14-4.08)
Model 4	1(control)	1.67(0.81-3.47)	1.12(0.51-2.44)	1.54(0.87-3.58)
Stroke	12(40%)	11(26.8%)	7(14.3%)	6(7.3%)
Unadjusted HR	1(control)	0.66(0.31-1.37)	0.27(0.11-0.71)	0.39(0.15-1.01)
Model 1	1(control)	0.737(0.35-1.56)	0.25(0.10-0.64)	0.41(0.16-1.08)
Model 2	1(control)	1.36(0.48-3.77)	0.28(0.09-0.84)	0.59(0.17-2.03)
Model 3	1(control)	1.27(0.44-3.68)	0.32(0.12-1.01)	0.73(0.19-2.80)
Model 4	1(control)	1.28(0.40-4.10)	0.34(0.09-1.26)	0.93(0.21-4.23)

*Model 1 was adjusted for age and gender. Model 2 was adjusted for the variables in model 1 plus hypertension, DM, AF, CHD, BMI, hemoglobin, plasma albumin, eGFR, LDL-C and HDL-C. Model 3 was adjusted for the variables in model 2 plus cardiovascular drugs. Model 4 was adjusted for the variables in model 3 plus LVEF, LAD and LVMI. NT-proBNP, N-terminal pro-brain natriuretic peptide; ACS: acute coronary syndrome; HR: hazard ratio; CI: confidence interval; CHD: coronary heart disease; HT: hypertension; DM: diabetes mellitus; AF: atrial fibrillation; BMI: body mass index; LDL-C: low density lipoprotein cholesterol; HDL-C: high density lipoprotein-cholesterol; eGFR: estimated glomerular filtration rate; LVEF: left ventricular ejection fraction; LAD: left atrial diameter; LVMI: left ventricular mass index; MACE: major adverse cardiovascular events.*

There were 202 patients with MACE during the follow-up. The incidence of MACE significantly increased from 16.6% in the lowest quartile of plasma NT-proBNP levels to 45.3% in the highest quartile of plasma NT-proBNP levels. A Kaplan-Meier survival analysis revealed significant differences between the groups (log-rank test, p = 0.002; Figure 2 ). The risk of MACE (HR, 1.77; 95% CI, 1.29–3.53; p = 0.04) for patients in the highest quartile of plasma NT-proBNP levels was significantly higher than for patients in the lowest quartile of plasma NT-proBNP levels, after adjusting for multiple cardiovascular risk factors. Further subgroup analysis found that the highest incidence of MACE was nonfatal acute coronary syndrome (ACS)(67.8%). The risk of ACS (HR, 1.89; 95%CI, 1.14-4.08; p=0.04) for patients in the highest quartile of plasma NT-proBNP levels was significantly higher than for patients in the lowest quartile of plasma NT-proBNP levels after adjusting for multiple cardiovascular risk factors (Model 3). However, plasma NT-proBNP levels were not associated with the risk of death (HR, 1.47; 95% CI, 0.88–2.45; p = 0.14) , MACE (HR, 1.31; 95% CI, 0.62–2.78; p = 0.48) or ACS (HR, 1.54; 95%CI, 0.87-3.58; p=0.20), according to results using the Cox proportional hazard regression model after further adjusting for LVEF, LAD, and LVMI (Model 4; Table 3 ).

**Figure 2:** – Kaplan-Meier curves demonstrating the cumulative incidence of MACE in the very elderly with different NT-proBNP levels (quartile 1: <124 pg/mL, quartile 2: 124-271 pg/mL, quartile 3: 271-668 pg/mL, and quartile 4: ≥668 pg/mL). The risk of MACE was significantly higher in quartile 4 (45.3%) than in quartile 1 (16.6%) (HR=1.77; 95%CI, 1.289-3.531; p=0.04). Log-rank, p=0.002. NT-proBNP: N-terminal pro-brain natriuretic peptide; HR: hazard ratio; CI: confidence interval.

### ROC curves of plasma NT-proBNP levels for predicting all-cause death and MACEs

The data shown in the ROC curves show that NT-proBNP is a reasonably accurate predictor of all-cause death and MACE. The area under the ROC curve was 0.71 (95% CI, 0.677–0.752; p < 0.001) for all-cause death ( Figure 3 ). The cut-off value for plasma NT-proBNP levels to predict all-cause death was 406 pg/mL and had a maximum Youden index of 0.36, with a sensitivity of 65% and a specificity of 81%. The area under the ROC curve was 0.58 (95% CI, 0.537–0.626; p = 0.001) for MACE ( Figure 4 ). The cut-off value for plasma NT-proBNP levels to predict MACE was 406 pg/mL and had a maximum Youden index of 0.23, with a sensitivity of 69% and a specificity of 54%.

**Figure 3:** – A ROC curve of NT-proBNP to predict the all-cause death. The AUC was 0.71 (95% CI, 0.677-0.752), p<0.001. ROC: receiver operating characteristic; NT-proBNP: N-terminal pro-brain natriuretic peptide; AUC: area under curve; CI: confidence interval.

**Figure 4:** – A ROC curve of NT-proBNP to predict MACE. The AUC was 0.58 (95% CI, 0.537-0.626), p=0.001. ROC: receiver operating characteristic; NT-proBNP: N-terminal pro-brain natriuretic peptide; AUC: area under the curve; CI: confidence interval.

## Discussion

The main finding of this study is that NT-proBNP is an independent predictor of all-cause death and MACE in this very elderly population, although the parameters of echocardiography weakened its predictive value. In addition, the risk of 5-year death (77.3%) and 5-year MACE (45.3%) was particularly increased in individuals with plasma NT-proBNP levels ≥668 pg/mL in this study, suggesting that it is possible to have an independent risk assessment with NT-proBNP in the elderly patients.

Many studies have confirmed that NT-proBNP is an important predictive biomarker in different populations,^1^ not only in patients with heart failure and other CVDs^14 , 15^ but also in the general population.^2 , 16^ However, in the very elderly, there are limited data on its prognostic predictive value. In the present study, NT-proBNP was an independent predictor of all-cause death and MACE in very elderly patients (≥80 years), which is consistent with results from previous studies.^4^ Vaes et al.^4^ first reported that NT-proBNP was an independent prognostic factor in the very elderly ( > 85 years). However, for that study specific population, a history of CVD was based on different diagnostic standards; not all participants underwent an echocardiography examination.^4^ These factors may affect the prognostic value of NT-proBNP.

In the present study, a history of hypertension, CHD, and AF were based on accepted diagnostic standards; all participants underwent an echocardiography examination. NT-proBNP was an independent predictor of all-cause death, MACE and ACS after adjusting for age, gender, and traditional cardiovascular risk factors. Since the prognostic value of NT-proBNP was no longer significantly present after adjusting for echocardiographic parameters (LVEF, LAD and LVMI), our hypothesis is that NT-proBNP measurement and echocardiography findings can complement each other. NT-proBNP measurement is a fast and inexpensive way of possibly preventing the need for an echocardiogram in case of low values and, on the other hand, it is a better indication for an echocardiogram in cases of the higher NT-proBNP level.

Currently, few studies have discussed the prognostic value of NT-proBNP in the very elderly, and there are no studies about the optimal cut-off value for plasma NT-proBNP levels to predict death or MACE in this population.

In previous studies, the optimal cut-off values differed for different populations,^17 , 18^ which were very high in patients with acute decompensated heart failure^19 , 20^ and <90 pg/mL in the general population.^16 , 18^ Fu et al.^17^ reported that the optimal cut-off value for NT-proBNP to predict death in older Chinese patients with coronary artery disease is 369.5 pg/mL in non-CKD patients and 2,584.1 pg/mL in CKD patients. In this study, the results from the ROC curves indicate that NT-proBNP is a reasonably accurate predictor of all-cause death and MACE. The areas under the ROC curves were 0.71 (95% CI, 0.677–0.752) for all-cause death and 0.58 (95% CI, 0.537–0.626) for MACE. The cut-off value for plasma NT-proBNP levels (406 pg/mL) had a sensitivity of 65% and a specificity of 81% to predict all-cause death, and a sensitivity of 69% and a specificity of 54% to predict MACE. But this value is not suitable as the optimal cut-off value to predict the all-cause death and MACE, because of low specificity and sensitivity. Simultaneously, it was also observed that the individuals at highest quartile (NT-proBNP level≥668pg/mL) had 77.3% risk of death and 45.3% risk of MACE during the follow-up period, significantly higher than the other three groups; this identifies high risk population and it is clinically relevant.

We think it is possible to have an independent risk assessment by assessing NT-proBNP levels in this elderly patients. It was very similar to an increased risk for cardiovascular morbidity and mortality observed by van Peet et al.^6^ found at the higher tertiles of NT-proBNP levels for men (cut-off level 649pg/mL), as well as at the higher tertiles of NT-proBNP levels for women (cutoff level 519pg/mL). They stated that high levels of NT-proBNP may help clinicians to identify patients who will probably benefit the most from a proactive follow-up, and our results were consistent with theirs.

This study has several limitations. First, only 45 (12.7%) patients died of cardiac causes in this study; most died of multiple-organ failure. Therefore, the predictive value of NT-proBNP for cardiac death was not analyzed in this study. Second, although the results were adjusted for multiple covariates that may be associated with plasma NT-proBNP levels, it is possible that residual confounding factors, such as tumors, pacemaker implantation, and silent myocardial ischemia, may impact the findings. Third, because of the long follow-up period, the primary cardiovascular drugs used may have changed with time and, thus, may not be reflected in the results of this study. Fourth, this study was performed at a single center in China, the population consisted of almost exclusively men, and all patients were hospitalized and very elderly, so the results cannot be applied to a broader population. Fifth, frailty and other physical parameters were not assessed in this study, which may impact the results. Sixth, the analysis of incidences of MACE did not consider a competing risk model with noncardiac death as competing risk, which may have underestimated the prognostic value of NT-proBNP for predicting MACE.

## Conclusion

NT-proBNP was identified as an independent predictor of all-cause death and MACE in hospitalized patients older than 80 years of age.
